# Combined multi‐metric assessment of diaphragm contractile function in healthy humans: Feasibility, validity and reliability

**DOI:** 10.1113/EP093294

**Published:** 2026-02-18

**Authors:** Camilla R. Illidi, Lee M. Romer

**Affiliations:** ^1^ Department of Sport, Health and Exercise Sciences, College of Health, Medicine and Life Sciences Brunel University of London Uxbridge UK

**Keywords:** carbon dioxide rebreathing, hypercapnia, inspiratory muscle, phrenic nerve stimulation, respiratory mechanics, ultrasound

## Abstract

The combined use of subcostal ultrasonography and respiratory manometry represents a novel, integrative method for quantifying diaphragm contractile function (force, velocity and power). We evaluated the technical feasibility, construct validity and within‐day test–retest reliability of this method during non‐volitional, volitional and reflexive respiratory perturbations in healthy adults. Two independent cohorts were studied. In Experiment 1 (*n *= 10), diaphragm excursion (subcostal ultrasonography) and transdiaphragmatic pressure (*P*_di_, manometry) were measured during unilateral magnetic phrenic nerve stimulation (non‐potentiated and potentiated twitches, paired stimuli at 10–100 Hz) and maximal sniffs. In Experiment 2 (*n *= 8), the same measurements were obtained during progressive CO_2_ rebreathing. All protocols were repeated after 20 min of rest. Diaphragm velocity and power were calculated as excursion/time and *P*_di_ × velocity, respectively. Ultrasound analysis was successful in >95% of cases. Potentiated twitches elicited greater *P*_di_, excursion and power than non‐potentiated twitches, with responses increasing at higher stimulation frequencies. Reliability improved with potentiation and high‐frequency stimulation and was moderate to excellent for peak responses during sniffs and CO_2_ rebreathing (ICC_3,_ *_k_* = 0.70–0.94) but poor for slope‐based measures (ICC_3,_ *_k_* ≤ 0.20). During CO_2_ rebreathing, excursion and velocity correlated strongly with inspiratory tidal volume (*r* = 0.83, *P* < 0.001) and mean inspiratory flow (*r* = 0.69, *P* < 0.001), respectively. These findings demonstrate that subcostal ultrasonography combined with manometry is a feasible, valid and reliable method for assessing diaphragm contractile function across non‐volitional, volitional and reflexive perturbations. With further refinement, this integrated method has translational potential for mechanistic research and clinical application.

## Abstract (highlights)

- **What is the central question of this study?**
  Does subcostal ultrasonography combined with respiratory manometry provide a feasible, valid and reliable means of assessing of diaphragm contractile properties (force, velocity and power) during non‐volitional, volitional and reflexive respiratory perturbations in healthy humans?
- **What is the main finding and its importance?**
  The combined method was technically feasible, demonstrated strong construct validity, and showed acceptable test–retest reliability for most indices during unilateral magnetic phrenic nerve stimulation, maximal sniffs and CO_2_ rebreathing. This method enables comprehensive assessment of diaphragm function across a range of contractile states and has translational potential for mechanistic research and clinical evaluation.

## 1 INTRODUCTION

The diaphragm is the principal muscle of inspiration (Grimby et al., 1976). Upon contraction, it descends, increasing thoracic volume and reducing intrathoracic pressure, thereby drawing air into the lungs. Beyond its ventilatory role, the diaphragm contributes to several non‐respiratory functions, including facilitation of venous return via modulation of thoraco‐abdominal pressure gradients, and generation of intra‐abdominal pressure to aid expulsive actions (e.g., coughing, defaecation and parturition) and maintain postural stability (Sheel & Romer, 2012). Impairment of this multifunctional muscle therefore may compromise both respiratory and non‐respiratory performance. Because diaphragm dysfunction is prevalent across a wide range of acute and chronic conditions (McCool & Tzelepis, 2012), accurate assessment of diaphragm function is essential for physiological research, clinical diagnosis, patient monitoring and targeted intervention.

Transdiaphragmatic pressure (*P*_di_), calculated as the difference between gastric and oesophageal pressures measured using catheter‐based manometry (*P*_di_ = *P*_ga_ − *P*_oe_), is regarded as the reference standard for assessing diaphragm function. The *P*_di_ responses to maximal voluntary efforts (e.g., sniffs) and non‐volitional perturbations (e.g., phrenic nerve stimulation, hypercapnia‐induced hyperpnoea) have been used widely to characterize the mechanical properties of the human diaphragm (Laveneziana et al., 2019). However, as a pressure‐derived index, *P*_di_ primarily reflects force generation and provides limited insight into overall contractile performance. A more complete evaluation requires assessment of power output, which accounts for both force output and shortening velocity (power = force × velocity).

Imaging modalities such as fluoroscopy, chest radiography, CT and MRI can provide detailed insights into diaphragm structure and motion but are constrained by radiation exposure, high cost and limited accessibility. In contrast, ultrasonography offers a practical, non‐invasive alternative that enables repeated assessment of diaphragm thickness, thickening and excursion (Dres et al., 2025; Laghi et al., 2021). Most combined ultrasound–manometry studies have used an intercostal approach to evaluate thickness‐based indices within the zone of apposition. However, such indices exhibit weak or inconsistent associations with *P*_di_ in both healthy individuals and clinical populations (Goligher et al., 2015; Poulard et al., 2022).

In contrast, the subcostal ultrasound approach, in which the transducer is positioned inferior to the costal margin, permits direct quantification of cranial–caudal excursion and derivation of shortening velocity (excursion/time). When combined with manometry, this approach enables estimation of diaphragm power output (*P*_di_ × velocity). To date, studies using this integrated method have typically acquired ultrasound and pressure data during separate manoeuvres while assuming conceptual equivalence between measures (Spiesshoefer et al., 2019, 2020). Such methodological separation precludes real‐time evaluation of pressure–motion coupling and neglects the intrinsic force–velocity relationship inherent to skeletal muscle.

Using the integrated subcostal‐manometry method, we previously demonstrated that diaphragm power is preserved during lower‐limb exercise through coordinated adjustments in shortening dynamics (Illidi & Romer, 2022). Subsequent work showed that inspiratory resistive loading reduces stimulation‐evoked diaphragm power primarily by constraining shortening velocity, with reductions in force playing a secondary role (Illidi & Romer, 2025). Despite these advances, ultrasound‐derived indices remain susceptible to operator dependence and motion artefact, particularly during rapid or high‐force contractions. Accordingly, a systematic appraisal of the feasibility, validity and reliability of this combined multi‐metric method is warranted.

The aim of the present study therefore was to investigate the combined use of subcostal ultrasonography and respiratory manometry for assessing diaphragm contractile function in healthy adults. The specific objectives were as follows: (1) to determine the technical feasibility of this method during phrenic nerve stimulation (non‐potentiated vs. potentiated, single vs. paired stimuli), maximal voluntary efforts (sniffs) and reflexively induced hyperpnoea (CO_2_ rebreathing); (2) to assess the construct validity of ultrasound‐derived indices of excursion, velocity and power across a range of contractile loads; and (3) to establish the within‐day test–retest reliability of these indices. Collectively, these objectives provide a comprehensive methodological framework to advance mechanistic understanding of diaphragm function and inform future translational applications in both research and clinical settings.

## 2 MATERIALS AND METHODS

### 2.1 Ethical approval

All procedures conformed to the *Declaration of Helsinki*, except for prior registration in a public database, and were approved by the Brunel University London Research Ethics Committee (8404‐A‐Aug/2018‐13769‐1 and 16371‐A‐Jul/2019‐19984‐1). Participants received detailed written and verbal information regarding study procedures, potential risks and anticipated benefits, and provided written informed consent.

### 2.2 Participants

Two independent groups of young, recreationally active adults with no history of smoking or cardiorespiratory disease volunteered to participate (Table 1). Eligibility criteria included age 18–40 years, body mass index 18.5–30.0 kg/m^2^ and pulmonary function within normal limits. All participants had previously taken part in our ultrasound investigations of diaphragm function (Illidi & Romer, 2022, 2025); the dataset presented herein is distinct from these prior analyses.

**TABLE 1:** Participant characteristics.

	Experiment 1 (5 ♁, 5 ♂)	Experiment 2 (8 ♂)
Anthropometrics		
Age, years	21 (2)	23 (7)
Stature, cm	1.67 (0.11)	1.80 (0.05)
Body mass, kg	66.3 (11.7)	77.1 (10.3)
Body mass index, kg/m^2^	23.5 (2.6)	23.7 (3.0)
Chest circumference, cm	82.9 (9.8)	94.4 (2.6)
Chest depth, cm	19.6 (2.3)	20.0 (1.1)
Chest width, cm	27.5 (2.7)	30.3 (1.7)
Pulmonary function		
TLC, L	5.97 (1.27) [100 (9)]	7.46 (0.87) [107 (10)]
RV, L	1.56 (0.36) [118 (27)]	1.86 (0.23) [112 (14)]
FRC_pleth_, L	3.16 (0.85) [104 (18)]	3.97 (0.72) [116 (18)]
FVC, L	4.71 (1.09) [108 (6)]	5.74 (0.47) [100 (6)]
FEV_1_, L	3.98 (0.84) [104 (8)]	4.75 (0.41) [101 (7)]
FEV_1_/FVC	0.84 (0.03) [97 (3)]	0.82 (0.05) [100 (4)]
MVV, L/min	147 (23) [94 (13)]	197 (28) [108 (14)]
PI_max_, cmH_2_O	−120 (25) [111 (21)]	−125 (16) [113 (15)]
PE_max_, cmH_2_O	156 (38) [121 (19)]	173 (19) [112 (11)]
Diaphragm function		
Thickness at FRC, mm	1.3 (0.2)	1.6 (0.5)
Thickness at TLC, mm	3.9 (0.9)	4.1 (0.7)
Thickening fraction, %	200 (53)	156 (40)
Maximal excursion, cm	6.35 (0.58)	6.78 (1.50)

*Note*: Data are means (SD). Values in square brackets are the percentage predicted.

Abbreviations: FRC_pleth_, plethysmography‐derived functional residual capacity; FVC, forced vital capacity; FEV_1_, forced expiratory volume in 1 s; MVV, maximal voluntary ventilation, estimated as FEV_1_ × 40; PE_max_, maximal static expiratory mouth pressure; PI_max_, maximal static inspiratory mouth pressure; RV, residual volume; TLC, total lung capacity.

### 2.3 Experimental overview

Each participant attended two laboratory sessions separated by 2–7 days and scheduled at the same time of day to minimize diurnal variability. Visit 1 comprised screening, baseline assessments and procedural familiarisation. Visit 2 comprised the experimental session, during which participants underwent either unilateral phrenic nerve stimulation followed by maximal voluntary sniffs (Experiment 1) or a CO_2_‐rebreathing protocol (Experiment 2). These perturbations were chosen to elicit a wide range of contractile loads and to test the robustness of ultrasound imaging in dynamic physiological conditions. To assess within‐day test–retest reliability, all procedures were repeated after 20 min of rest. Participants refrained from strenuous physical exercise and alcohol for ≥24 h, caffeine for ≥12 h and large meals for ≥3 h before each visit.

### 2.4 Baseline characteristics and familiarization

Anthropometric, pulmonary and diaphragm characteristics were assessed using previously described protocols (Illidi & Romer, 2022). Chest circumference, depth and width were measured at relaxation volume [functional residual capacity (FRC)]. Spirometry and body plethysmography (MasterScreen PFT, CareFusion, Hoechberg, Germany) were conducted in accordance with international recommendations (Graham et al., 2019; Miller et al., 2005; Wanger et al., 2005). Maximal inspiratory and expiratory mouth pressures (PI_max_ and PE_max_) were obtained from residual volume (RV) and total lung capacity (TLC), respectively, using a hand‐held manometer (MicroRPM, CareFusion) (Laveneziana et al., 2019). Values were reported as absolute and percentage predicted (Evans & Whitelaw, 2009; Quanjer et al., 2012; Stocks & Quanjer, 1995). Diaphragm thickness was measured at FRC and TLC using intercostal ultrasonography, with the thickening fraction calculated from FRC to TLC, whereas maximal excursion was assessed over the same lung volumes via a subcostal approach (Laursen et al., 2021). Finally, participants were thoroughly familiarized with the experimental procedures, excluding catheter placement.

### 2.5 Experiment 1

Diaphragm contractile function was assessed using magnetic stimulation of the right phrenic nerve. Unilateral stimulation was selected to facilitate paired stimulation and reduce the likelihood of submaximal activation, the latter of which might underestimate contractile function (Angus et al., 2023). Stimulation was delivered using a figure‐of‐eight coil (D70 Alpha B.I., Magstim, Whitland, UK), powered by two magnetic stimulators (Magstim 200, Magstim) via a paired‐pulse module (BiStim, Magstim) (Mills et al., 1995). Participants were tested in a semi‐recumbent position (30° hip angle, legs extended, arms resting by the torso) to optimize ultrasound image acquisition. The optimal coil position was identified along the anterior border of the right sternocleidomastoid at 70% stimulator output and marked to ensure consistent placement. Stimulations were delivered at FRC, confirmed via end‐expiratory oesophageal pressure, with participants maintaining a closed glottis. An incremental stimulation protocol was used to confirm supramaximal stimulation, defined as a plateau in twitch transdiaphragmatic twitch (*P*_di,tw_) despite further increases in stimulator output. Subsequent stimulations were conducted at 100% output. The experimental protocol comprised: (1) five non‐potentiated single twitches (1 Hz); (2) three paired stimuli at 10, 50 and 100 Hz (interstimulus intervals of 100, 20 and 10 ms); (3) five maximal voluntary sniffs from FRC, each separated by 30 s and performed as ‘short, sharp sniffs as hard as possible’ (Miller et al., 1985); and (4) five maximal Müller‐expulsive manoeuvres (2–3 s efforts against a semi‐occluded mouthpiece), each followed ∼5 s later by a potentiated single twitch (Mador et al., 1994; Wragg et al., 1994).

### 2.6 Experiment 2

Diaphragm contractile function during reflexive increases in ventilation was assessed using a CO_2_‐rebreathing protocol (Read, 1967). Participants rested in semi‐Fowler's position for 10 min to establish a relaxed, wakeful state. They then wore a nose‐clip and breathed for 3 min through a mouthpiece–valve assembly (2110, Hans Rudolph, Shawknee, KS, USA; 53.5 mL dead space). After exhalation to RV, the valve was closed, and participants equilibrated with the rebreathing circuit by completing three deep, rapid inspirations from a latex reservoir bag (volume ≈ vital capacity + 1 L) filled with 95% O_2_ and 5% CO_2_ (BOC, Guilford, UK). Participants were instructed to close their eyes, relax, and breathe as needed until end‐tidal CO_2_ ($P_{\mathrm{ET},CO_{2}}$) reached 55 mmHg, at which point the valve was reopened. To minimize variability attributable to arousal or behavioural input (Spengler & Shea, 2001), testing was conducted in a quiet laboratory, with minimal experimenter–participant interaction.

### 2.7 Diaphragm shortening

**FIGURE 1:**
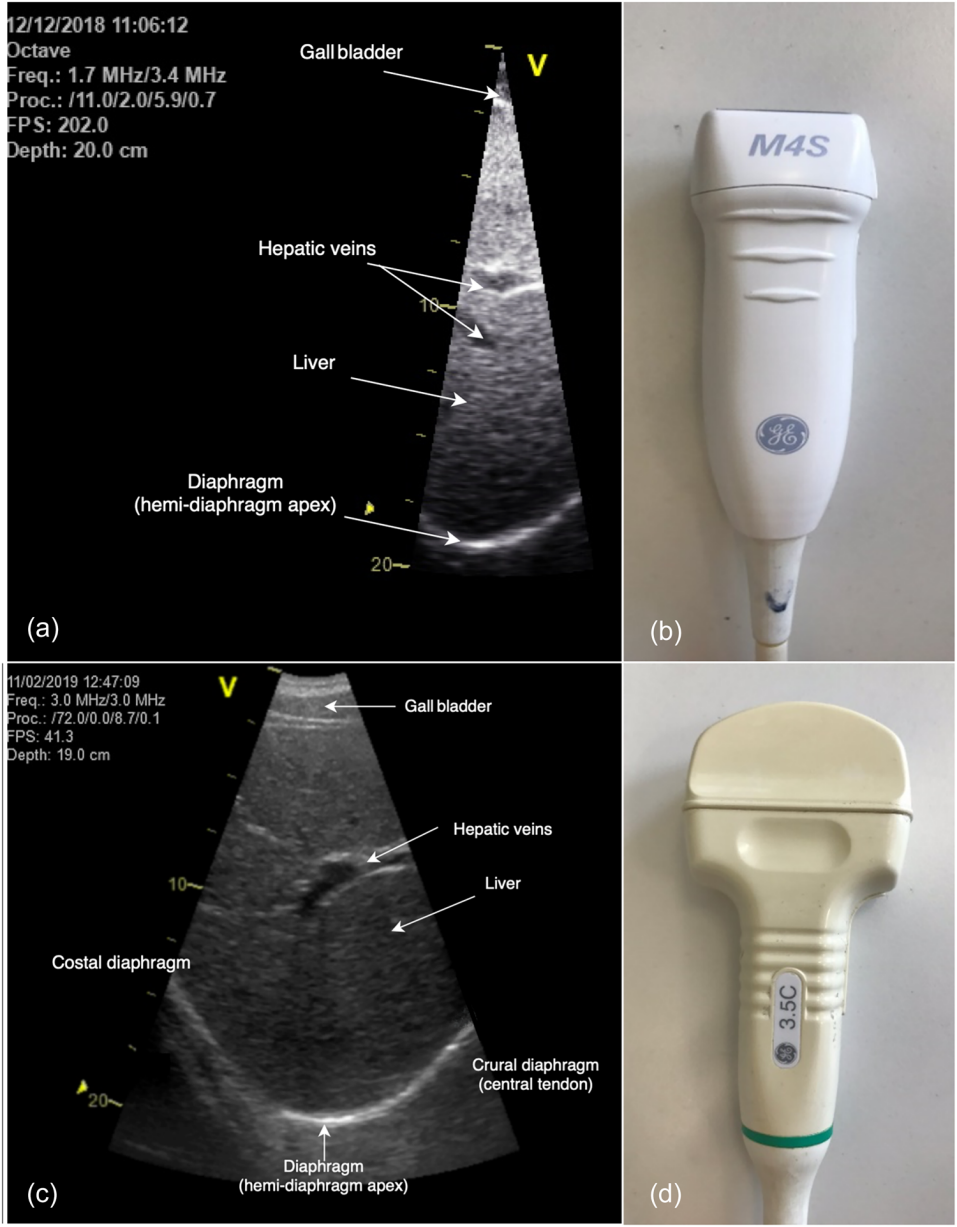
Representative B‐mode ultrasound images of the right crural hemidiaphragm at relaxation volume in a female participant. Images were obtained using a phased‐array transducer (a, b) and a curved‐array transducer (c, d).

Ultrasound procedures were performed as previously described (Illidi & Romer, 2022, 2025). In brief, diaphragm excursion was recorded using a Vivid 7 Pro ultrasound system (GE Medical, Horten, Norway). In Experiment 1, a phased‐array transducer (1.5–4.0 MHz, M3S, GE Medical) was used to provide a narrow field of view centred on the crural apex, with a penetration depth of 200–250 mm and an acquisition frame rate of 200–220 Hz (Figure 1a, b). In Experiment 2, a curved‐array transducer (2.4–5.0 MHz, 3.5C, GE Medical) provided a wider field of view, with an adequate frame rate (40–60 Hz) for the slower breathing dynamics characteristic of CO_2_ rebreathing (Figure 1c, d).

For both experiments, the transducer was positioned below the costal margin, between the right midclavicular and anterior axillary lines (Laursen et al., 2021). One focal point was placed at the diaphragm position at relaxation volume to optimize lateral resolution, with the inferior vena cava serving as a landmark during CO_2_ rebreathing. To ensure consistency in probe placement, the transducer site was marked with indelible ink, and the sonographer's arm was stabilized using a foam support. Skin surface electrodes (3M, Bracknell, UK) were positioned bilaterally along the mid‐axillary line to synchronize image acquisition with chest wall motion. All images were acquired in B‐mode and processed offline using angle‐independent anatomical M‐mode (EchoPac v.6.1, GE Medical; Orde et al., 2016). Cine loops were analysed only when hyperechoic lines clearly delineated the onset and termination of contraction. Diaphragm excursion and excursion time were measured using digital callipers as the amplitude of displacement and the time interval from onset to peak excursion, respectively. To ensure procedural consistency and minimize measurement variability, all ultrasound images were acquired and analysed by a single experienced sonographer (C.R.I.). Frame rates exceeded Nyquist sampling requirements by ∼10‐fold, ensuring accurate capture of rapid contractions and minimizing aliasing or temporal distortion.

### 2.8 Respiratory pressures

In Experiment 1, *P*_oe_ and *P*_ga_ were measured using balloon‐tipped catheters (CooperSurgical, Berlin, Germany). Prior to catheter placement, 1 mL of 2% lignocaine hydrochloride was applied to the nasal mucosa for topical anaesthesia. Each catheter was passed pernasally, advanced into the stomach and connected via short rigid tubing to a differential pressure transducer (DP45‐3, Validyne, Northridge, CA, USA). The transducers were calibrated against an electro‐manometer (C9553, JMW, Harlow, UK). Balloon volumes were adjusted using a glass syringe (1 mL air for oesophageal, 2 mL for gastric), following a brief Valsalva manoeuvre to ensure deflation. The oesophageal catheter was withdrawn until inspiration elicited a negative *P*_oe_ deflection, after which it was retracted an additional ∼10 cm such that the distal tip was in the lower third of the oesophagus. In Experiment 2, *P*_oe_ and *P*_ga_ were measured using a custom catheter with integrated micro‐transducers (Gaeltec, Dunvegan, Isle of Skye, UK). Calibration was performed by placing the catheter in a sealed air‐filled tube connected to the electro‐manometer, with voltage outputs adjusted over the physiological range (Tiller et al., 2017). In both experiments, pressure signals were amplified (Experiment 1: CD280, Validyne; Experiment 2: 1902, Cambridge Electronic Design, Cambridge, UK), digitized at 200 Hz (Micro1401mk‐II, Cambridge Electronic Design) and recorded online (Spike2, Cambridge Electronic Design). In each experiment, correct catheter positioning was confirmed using the dynamic occlusion test (Baydur et al., 1982), after which the catheter was secured at the nares with adhesive tape. Instantaneous transdiaphragmatic pressure (*P*_di_) was calculated as the difference between gastric and oesophageal pressures (*P*_di_ = *P*_ga_ − *P*_oe_).

### 2.9 Ventilation and gas exchange

Ventilatory and pulmonary gas‐exchange responses were assessed breath‐by‐breath using an online system comprising a turbine flowmeter, sample line and fast‐response O_2_ and CO_2_ analysers (Oxycon Pro, Viasys, Hoechberg, Germany). Variables included inspiratory minute ventilation ($\dot{V}$_I_), inspiratory tidal volume (*V*_TI_), respiratory frequency (*f*_R_), inspiratory time (*t*_I_), ratio of *t*_I_ to total inspiratory time (*t*_I_/*t*_TOT_), mean inspiratory flow (*V*_TI_/*t*_I_) and $P_{\mathrm{ET},CO_{2}}$. The turbine and gas analysers were calibrated immediately before each trial using a 3 L syringe and precision‐analysed gas mixtures, respectively. All signals were integrated with the primary data acquisition system via an external analog‐to‐digital interface (DAQ‐30A16, Eagle Technology, Cape Town, South Africa).

### 2.10 Data processing and analysis

For single and paired stimuli, *P*_di,tw_ was defined as the pressure swing from end‐expiration to peak. Sniff pressure (*P*_di,sn_) was calculated using the same approach. Twitch and sniff responses (*P*_di_, excursion, velocity and power) were expressed as the mean of the final three single twitches and maximal sniffs and the mean of all three paired stimuli. For CO_2_ rebreathing, ventilatory and pressure data were analysed breath by breath after exclusion of artefacts, such as swallows, coughs, sighs or flow not crossing zero. Tidal *P*_di_ was defined as the active component of mean inspiratory pressure ($\bar{P}$_di_) (Illidi & Romer, 2022). Ultrasound cine loops (B‐mode) were recorded twice at baseline and at 30 s intervals thereafter. Individual breaths captured within each cine loop were identified in the acquisition system and averaged over 15 s (30 s at rest). The final 15 s of CO_2_ rebreathing was designated the peak response. For all perturbations, excursion velocity was calculated as excursion divided by contraction time, and diaphragm power was calculated as the product of pressure (*P*_di,tw_ or $\bar{P}$_di_) and excursion velocity (Illidi & Romer, 2022, 2025).

### 2.11 Statistics

Statistical analyses were conducted using SPSS (v.26.0, IBM Corp., Armonk, NY, USA) and GraphPad Prism (v.9.3, GraphPad Software, San Diego, CA, USA). Data normality and homogeneity of variance were confirmed using the Shapiro–Wilk test and Levene's test, respectively. Technical feasibility was quantified as the proportion of ultrasound images that provided clear and stable visualization of diaphragm motion. In Experiment 1, construct validity was evaluated by comparing non‐potentiated and potentiated single‐twitch responses using Student's paired *t*‐tests, and by analysing frequency responses (1–100 Hz) using mixed ANOVA with Benjamini–Hochberg correction for multiple comparisons (Curran‐Everett, 2000). Mauchly's test was used to assess sphericity, and when violated, degrees of freedom were corrected using the Greenhouse–Geisser method. Effect sizes were reported as Cohen's *d*_z_ (0.20 = small, 0.50 = medium and 0.80 = large) and partial eta squared ($\eta_{p}^{2}$; 0.001 = small, 0.06 = medium and 0.14 = large) (Lakens, 2013). In Experiment 2, slopes for ventilatory and pressure responses relative to $P_{\mathrm{ET},CO_{2}}$ were calculated from breath‐by‐breath data using least‐squares regression, with a single linear segment applied due to the intentional omission of hyperventilation prior to rebreathing. Ultrasound‐derived indices (excursion, time, velocity and power) were analysed as 15 s mean values regressed against time‐matched $P_{\mathrm{ET},CO_{2}}$. Individual slopes were estimated for each participant and trial, and mean differences in slope (Trial 1 ‐ Trial 2) were analysed using paired *t*‐tests (Zar, 2014). Peak responses were also compared between trials using paired *t*‐tests. Construct validity was examined further by regressing diaphragm excursion and excursion velocity against tidal volume (*V*_TI_) and mean inspiratory flow (*V*_TI_/*t*_I_), respectively; the strength of these relationships was expressed as Pearson's correlation coefficients.

Test–retest reliability was evaluated using the standard error of measurement (SE_M_), coefficient of variation (CV) and intraclass correlation coefficient (ICC). The SE_M_, reflecting absolute precision, was calculated as SD × √(1 − *r*), where SD is the standard deviation of observed values and *r* the ICC (Bruton et al., 2000). Relative variability was expressed as CV (%) using the root mean square method for paired measurements (Hyslop & White, 2009): 100 × **√**[Σ(*d*/*m*)^2^/2*n*], where *d* is the difference, *m* the mean of paired measurements, and *n* the number of data pairs. The standard error of the CV (SE_CV_) was calculated as *s*/**√** *n*, where *s* is the standard deviation of CV values, and 95% confidence intervals (CI) were estimated as mean CV ± 1.96 × SE_CV_ (Bland, 2006). The ICCs and 95% confidence intervals were calculated using a mean‐rating (*k* = 2), absolute‐agreement, two‐way mixed‐effects model [ICC(3,*k*)] (Koo & Li, 2016) and interpreted as poor (<0.50), moderate (0.50–0.75), good (0.75–0.90) or excellent (>0.90) reliability. Descriptive statistics are reported as the mean (SD). Statistical significance was accepted at *P* ≤ 0.05 (two‐tailed).

## 3 RESULTS

### 3.1 Experiment 1

#### 3.1.1 Technical feasibility

**FIGURE 2:**
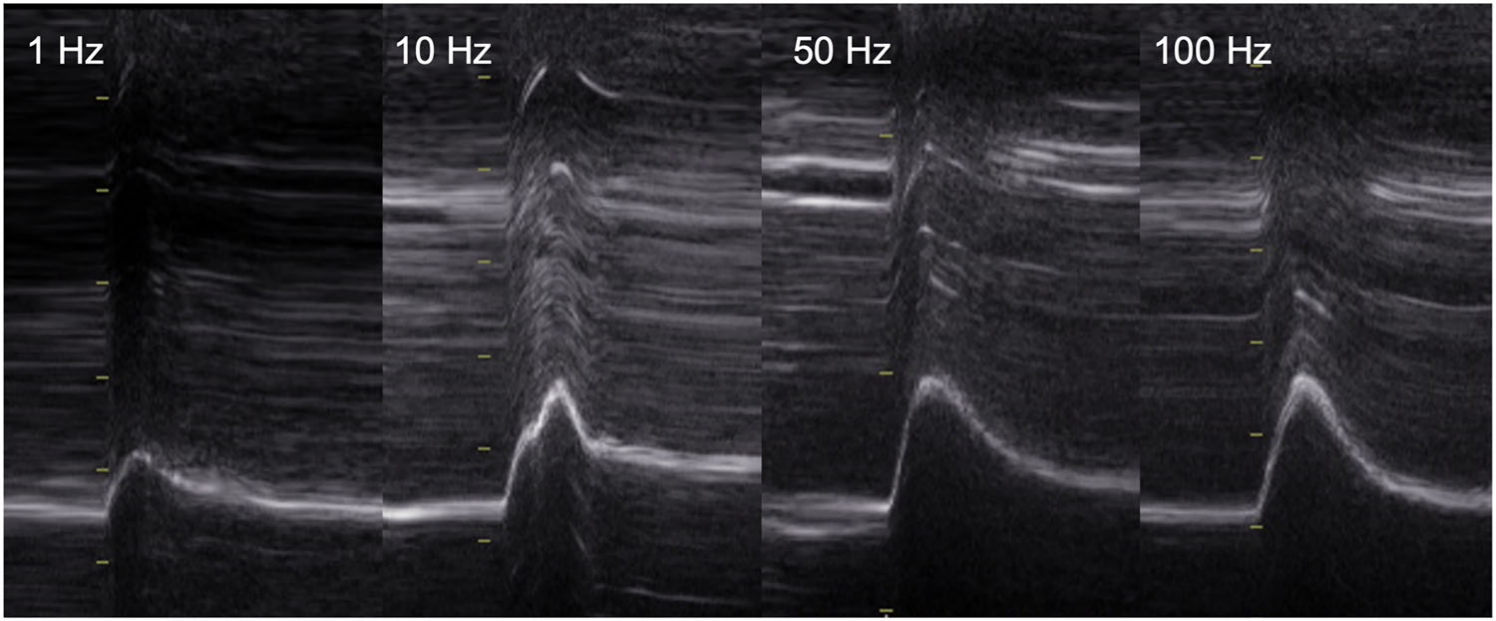
Representative anatomical motion‐mode (AM‐mode) ultrasound images of the right crural hemidiaphragm in response to single (1 Hz) and paired (10, 50 and 100 Hz) unilateral magnetic phrenic nerve stimulation in a female participant. Diaphragm excursion increased progressively with stimulation frequency (1.10 cm at 1 Hz; 2.10 cm at 100 Hz). Axis scales are identical across panels.

Clear and stable images were obtained in 95%–100% of evoked twitches and in 97% of voluntary sniffs, enabling successful analysis in nearly all cases. Representative images of crural diaphragm excursion in response to single‐ and paired‐stimuli are shown in Figure 2.

#### 3.1.2 Construct validity

**FIGURE 3:**
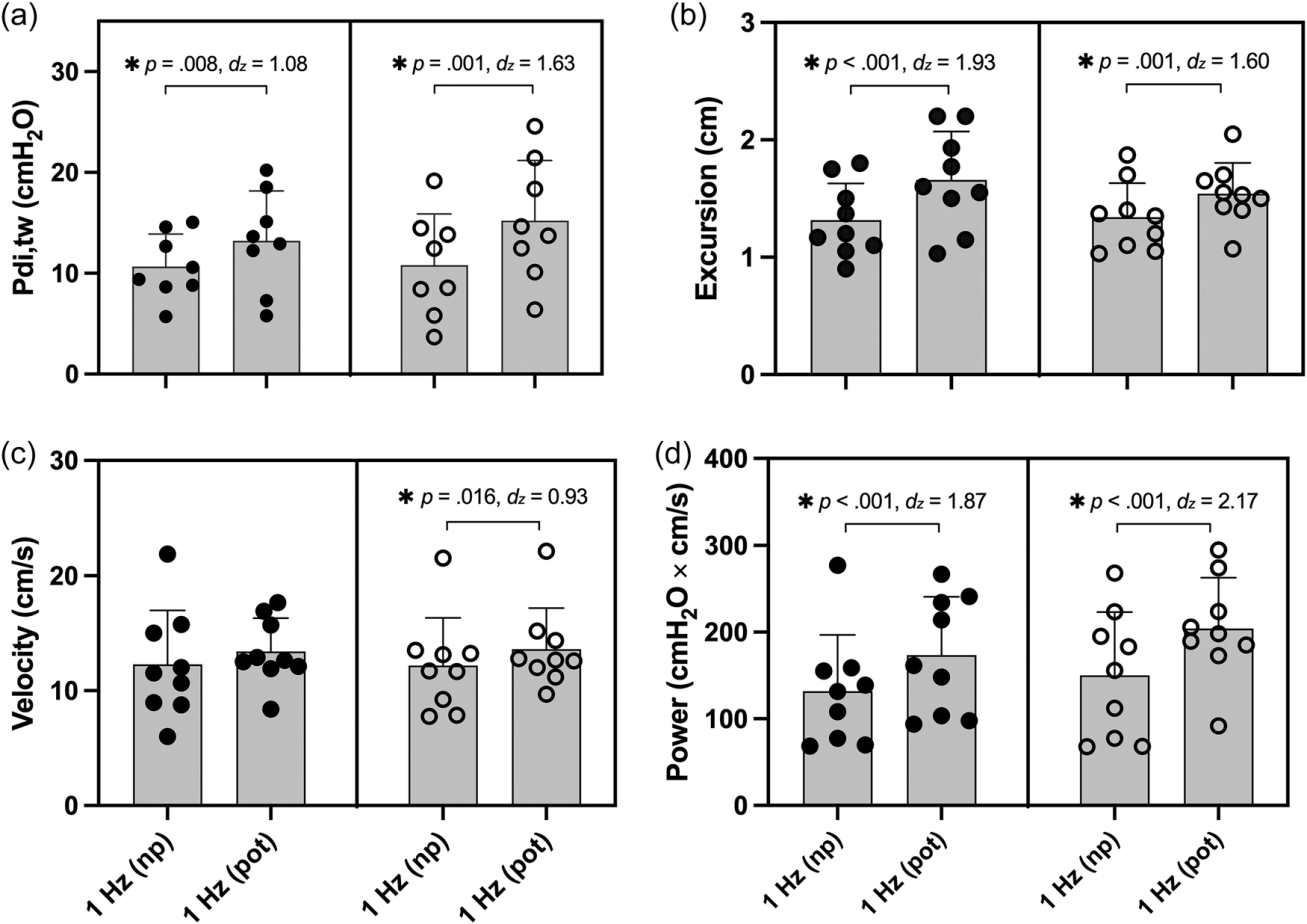
Group diaphragm contractile responses to supramaximal non‐potentiated and potentiated single‐twitch stimulation. Bars represent the mean (SD) for 10 participants, with individual data from Trial 1 (filled symbols) and Trial 2 (open symbols). *P < 0.05. Abbreviations: 1 Hz (np), non‐potentiated twitch; 1 Hz (pot), potentiated twitch; *P*_di,tw_, twitch transdiaphragmatic pressure.

Single‐twitch responses are presented in Figure 3. Potentiated twitches elicited significantly greater *P*_di_ (+28%), excursion (+27%) and power (+59%) compared with non‐potentiated twitches, with large effect sizes (*d*_z_ = 0.93–2.17). Excursion time increased modestly (+6%), but did not reach statistical significance, resulting in a 17% increase in excursion velocity that achieved significance only in Trial 2.

**FIGURE 4:**
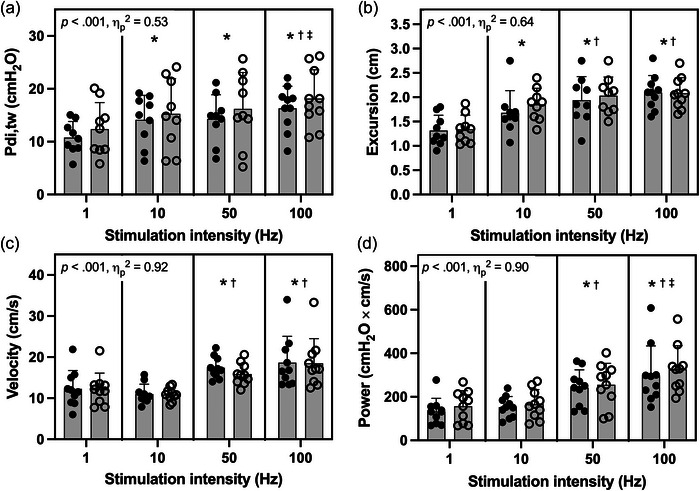
Group diaphragm contractile responses to supramaximal single‐twitch (1 Hz) and paired phrenic nerve stimulation (10, 50 and 100 Hz). Bars represent the mean (SD) for 10 participants, with individual data plotted from Trial 1 (filled symbols) and Trial 2 (open symbols). Stimulation frequency elicited significant main effects (statistical parameters shown in each panel). Pairwise differences: *P < 0.05 vs. 1 Hz; †*P *< 0.05 vs. 10 Hz; ‡*P *< 0.05 vs. 50 Hz. Abbreviations: *P*_di,tw_, twitch transdiaphragmatic pressure.

Frequency–response relationships are illustrated in Figure 4. Both *P*_di,tw_ and excursion increased progressively with stimulation frequency, with excursion reaching a plateau at 50 Hz. In contrast, excursion velocity and power remained stable between 1 and 10 Hz but increased sharply at 50 and 100 Hz (+57%–76% and +67%–110%, respectively; both *P* < 0.001). These changes were partly attributable to reductions in contraction time (−14% from 10 to 50 Hz, *P* < 0.001; −18% from 10 to 100 Hz, *P* = 0.063). Overall, higher stimulation frequencies produced graded increases in force, excursion, velocity and power, with contractile indices (velocity and power) exhibiting the strongest frequency dependence.

#### 3.1.3 Test–retest reliability

Reliability estimates are summarized in Tables 2, 3, 4. For non‐potentiated twitches, excursion time demonstrated ‘good’ reliability (ICC = 0.84, CV = 0.22%), whereas other indices were less consistent. Measurement reliability improved with potentiation: excursion velocity achieved ‘excellent’ reliability (ICC = 0.94, CV = 0.18%), while excursion and excursion time were classified as ‘good’ (ICCs = 0.76 and 0.74). In contrast, *P*_di,tw_ and power exhibited only ‘moderate’ reliability (ICCs = 0.63). Reliability was enhanced further with paired stimulation. At 100 Hz, excursion velocity demonstrated ‘excellent’ reliability (ICC = 0.94), whereas excursion, excursion time and power were classified as ‘good’. Across all stimulation frequencies, *P*_di,tw_ consistently demonstrated ‘moderate’ reliability. For voluntary sniffs, excursion time showed ‘excellent’ reliability, *P*_di_ and power demonstrated ‘good’ reliability,, and excursion and velocity were classified as ‘moderate’. Overall, potentiation, high‐frequency stimulation and volitional efforts yielded the most reliable measurements, particularly for ultrasound‐derived indices.

**TABLE 2:** Within‐day, between‐trial reliability of diaphragm responses to non‐potentiated and potentiated single‐twitch contractions.

Parameter	Trial 1	Trial 2	SE_M_	CV (%)	ICC	*P*‐value
1 Hz (np)						
*P*_di,tw_, cmH_2_O	10.8 (2.9)	11.6 (5.4)	0.47	1.90 (1.07–2.73)	0.39 (−0.32–0.81)	0.130
Excursion, cm	1.32 (0.30)	1.22 (0.47)	0.056	1.99 (1.12–2.86)	0.38 (−0.30–0.80)	0.131
Excursion time, s	0.114 (0.026)	0.115 (0.022)	0.000	0.22 (0.12–0.31)	0.84 (0.47–0.96)	**0.001**
Excursion velocity, cm/s	12.3 (4.5)	11.2 (5.3)	0.59	2.38 (1.33–3.42)	0.45 (−0.23–0.83)	0.092
Power, cmH_2_O×cm/s	118 (73)	123 (78)	3.0	1.17 (0.66–1.68)	0.42 (−0.31–0.82)	0.114
1 Hz (pot)						
*P*_di,tw_, cmH_2_O	13.1 (4.4)	15.6 (5.5)	1.07	4.35 (2.44–6.25)	0.63 (0.09–0.89)	**0.009**
Excursion, cm	1.67 (0.39)	1.55 (0.25)	0.041	1.86 (1.05–2.68)	0.76 (0.30–0.94)	**0.001**
Excursion time, s	0.124 (0.017)	0.119 (0.020)	0.020	1.03 (0.58–1.48)	0.74 (0.29–0.93)	**0.004**
Excursion velocity, cm/s	13.8 (3.3)	13.7 (3.4)	0.02	0.18 (0.10–0.26)	0.94 (0.79–0.99)	**<0.001**
Power, cmH_2_O×cm/s	179 (71)	204 (56)	10.8	3.27 (1.84–4.71)	0.63 (0.10–0.89)	**0.014**

*Note*: Data for Trial 1 and Trial 2 are means (SD) for 10 participants. Data for CV and ICC are means (95% CI). No group mean differences were observed between Trial 1 and Trial 2 (*P* > 0.05). All *P*‐values reflect statistical testing of the ICCs against zero reliability, with values in bold representing *P* ≤ 0.05.

Abbreviations: CV, coefficient of variation; ICC, intraclass correlation coefficient; np, non‐potentiated; pot, potentiated.; *P*_di,tw_, twitch transdiaphragmatic pressure; SE_M_, standard error of measurement.

**TABLE 3:** Within‐day, between‐trial reliability of diaphragm responses to paired stimuli (10, 50 and 100 Hz).

Parameter	Trial 1	Trial 2	SE_M_	CV (%)	ICC	*P*‐value
10 Hz						
*P*_di,tw_, cmH_2_O	14.1 (4.4)	15.0 (6.3)	0.39	1.58 (0.89–2.27)	0.64 (0.06–0.90)	**0.019**
Excursion, cm	1.68 (0.43)	1.85 (0.31)	0.059	2.41 (1.35–3.46)	0.76 (0.20–0.94)	**0.001**
Excursion time, s	0.11 (0.03)	0.17 (0.02)	0.038	10.00 (5.62–14.38)	0.11 (−0.07–0.48)	0.091
Excursion velocity, cm/s	11.0 (2.4)	10.9 (1.6)	0.047	0.21 (0.12–0.30)	0.47 (−0.25–0.84)	0.088
Power, cmH_2_O×cm/s	150 (44)	159 (61)	5.0	1.38 (0.77–1.98)	0.32 (−0.41–0.78)	0.187
50 Hz						
*P*_di,tw_, cmH_2_O	14.3 (4.3)	16.3 (6.5)	0.72	3.28 (1.84–4.71)	0.74 (0.28–0.93)	**0.003**
Excursion, cm	1.99 (0.32)	2.09 (0.31)	0.027	1.23 (0.69–1.76)	0.85 (0.46–0.96)	**0.000**
Excursion time, s	0.11 (0.02)	0.13 (0.02)	0.010	4.10 (2.30–5.89)	0.54 (−0.10–0.88)	**0.005**
Excursion velocity, cm/s	18.0 (2.2)	16.4 (2.2)	0.86	2.32 (1.30–3.34)	0.42 (−0.11–0.80)	0.054
Power, cmH_2_O×cm/s	255 (76)	262 (96)	2.3	0.63 (0.36–0.91)	0.75 (0.26–0.93)	**0.005**
100 Hz						
*P*_di,tw_, cmH_2_O	16.4 (4.2)	17.9 (5.5)	0.75	2.26 (1.27–3.25)	0.53 (−0.08–0.86)	**0.047**
Excursion, cm	2.10 (0.35)	2.09 (0.32)	0.003	0.12 (0.07–0.17)	0.82 (0.41–0.95)	**0.002**
Excursion time, s	0.12 (0.02)	0.11 (0.02)	0.002	1.08 (0.61–1.56)	0.75 (0.30–0.93)	**0.004**
Excursion velocity, cm/s	19.2 (6.3)	19.4 (5.6)	0.049	0.35 (0.20–0.50)	0.93 (0.76–0.98)	**<0.001**
Power, cmH_2_O×cm/s	337 (104)	312 (132)	8.6	1.95 (1.10–2.81)	0.77 (0.40–0.94)	**0.002**

*Note*: Data for Trial 1 and Trial 2 are means (SD) for 10 participants. Data for CV and ICC are means (95% CI). No group mean differences were observed between Trial 1 and Trial 2 (*P* > 0.05). All *P*‐values reflect statistical testing of the ICCs against zero reliability, with values in bold representing *P* ≤ 0.05.

Abbreviations: CV, coefficient of variation; ICC, intraclass correlation coefficient; *P*_di,tw_, twitch transdiaphragmatic pressure; SE_M_, standard error of measurement.

**TABLE 4:** Within‐day, between‐trial reliability of diaphragm responses to maximal sniffs.

Parameter	Trial 1	Trial 2	SE_M_	CV (%)	ICC	*P*‐value
*P*_di,sn_, cmH_2_O	80.8 (36.0)	77.4 (32.3)	1.18	1.07 (0.60–1.54)	0.76 (0.29–0.94)	**0.004**
Excursion, cm	1.80 (0.62)	2.09 (0.50)	0.113	3.73 (2.09–5.36)	0.70 (0.18–0.91)	**0.004**
Excursion time, s	0.22 (0.11)	0.23 (0.12)	0.001	0.33 (0.19–0.48)	0.94 (0.76–0.98)	**<0.001**
Excursion velocity, cm/s	10.8 (6.4)	10.8 (5.3)	0.020	0.12 (0.07–0.17)	0.67 (0.09–0.91)	**0.015**
Power, cmH_2_O×cm/s	922 (801)	918 (698)	1.3	0.11 (0.06–0.16)	0.79 (0.35–0.95)	**0.002**

*Note*: Data for Trial 1 and Trial 2 are means (SD) for 10 participants. Data for CV and ICC are means (95% CI). No group mean differences were observed between Trial 1 and Trial 2 (*P* > 0.05). All *P*‐values reflect statistical testing of the ICCs against zero reliability, with values in bold representing *P* ≤ 0.05.

Abbreviations: CV, coefficient of variation; ICC, intraclass correlation coefficient; *P*_di,sn_, sniff transdiaphragmatic pressure; SE_M_, standard error of measurement.

### 3.2 Experiment 2

#### 3.2.1 Ventilatory and pressure responses

Slope and peak data are summarized in Tables 5 and 6, respectively. The CO_2_‐rebreathing protocol was well tolerated, with no adverse events. Resting $P_{\mathrm{ET},CO_{2}}$ did not differ significantly between trials (Trial 1, 39.2 ± 3.7 mmHg; Trial 2, 38.0 ± 5.1 mmHg, *P* = 0.106). Each trial was terminated at a consistent $P_{\mathrm{ET},CO_{2}}$ (55.6 ± 0.2 mmHg). The rate of CO_2_ rise was within the expected range (3–6 mmHg/min; Read, 1967) and was comparable between trials. Trial durations were also similar (2.48 ± 0.70 vs. 2.99 ± 1.37 min, *P* = 0.097).

**TABLE 5:** Within‐day, between‐trial reliability of slope responses to CO_2_‐rebreathe.

Parameter	Trial 1	Trial 2	SE_M_	CV (%)	ICC	*P*‐value
Ventilatory indices						
$rrP_{\mathrm{ET},CO_{2}}$, mmHg/min	5.15 (1.14)	4.60 (0.86)	0.136	1.46 (0.82–−2.10)	0.88 (0.05–0.98)	**0.001**
$\dot{V}$_I_ vs. $P_{\mathrm{ET},CO_{2}}$, L/min/mmHg	1.65 (0.73)	1.79 (1.47)	0.059	9.16 (5.14–13.17)	0.64 (−0.82–0.93)	**0.017**
*V*_TI_ vs. $P_{\mathrm{ET},CO_{2}}$, mL/mmHg	85 (60)	68 (65)	6.0	1.76 (0.99–2.53)	0.76 (0.26–0.95)	**0.010**
*f*_R_ vs. $P_{\mathrm{ET},CO_{2}}$, breaths/mmHg	0.06 (0.43)	0.24 (0.48)	0.051	15.4 (8.7–22.2)	0.47 (−0.23–0.86)	0.099
*t*_I_ vs. $P_{\mathrm{ET},CO_{2}}$, s/mmHg	−0.048 (0.082)	−0.068 (0.130)	0.0072	20.7 (11.6–29.8)	0.73 (0.14–0.94)	**0.015**
Inspiratory pressures						
$\;\bar{P}$_di_ vs. $P_{\mathrm{ET},CO_{2}}$, cmH_2_O/mmHg	0.53 (0.43)	0.39 (0.30)	0.037	7.07 (3.97–10.16)	0.81 (0.18–0.96)	**0.017**
$\;\bar{P}$_oe_ vs. $P_{\mathrm{ET},CO_{2}}$, cmH_2_O/mmHg	−0.43 (0.18)	−0.32 (0.25)	0.035	4.67 (2.62–6.71)	0.93 (0.65–0.99)	**0.002**
$\;\bar{P}$_ga_ vs. $P_{\mathrm{ET},CO_{2}}$, cmH_2_O/mmHg	0.19 (0.15)	0.18 (0.13)	0.0032	2.70 (1.52–3.89)	0.80 (0.11–0.96)	**0.015**
Diaphragm shortening						
Excursion vs. $P_{\mathrm{ET},CO_{2}}$, cm/mmHg	0.125 (0.039)	0.108 (1.07)	0.0112	2.58 (1.45–3.71)	0.10 (−0.67–0.73)	0.406
Excursion velocity vs. $P_{\mathrm{ET},CO_{2}}$, cm/s/mmHg	0.062 (0.023)	0.051 (0.035)	0.0070	5.15 (2.89–7.41)	0.20 (−0.41–0.75)	0.286
Power vs. $P_{\mathrm{ET},CO_{2}}$, cmH_2_O×cm/s/mmHg	0.338 (0.314)	0.512 (0.700)	0.1185	22.7 (12.8–32.7)	0.07 (−0.75–0.72)	0.454

*Note*: Data for Trial 1 and Trial 2 are means (SD) for eight participants. Data for CV and ICC are means (95% CI). No group mean differences were observed between Trial 1 and Trial 2 (*P* > 0.05). All *P*‐values reflect statistical testing of the ICCs against zero reliability, with values in bold representing *P* ≤ 0.05.

Abbreviations: CV, coefficient of variation; *f*_R_, respiratory frequency; ICC, intraclass correlation coefficient $\bar{P}$_di_, mean inspiratory transdiaphragmatic pressure; $P_{\mathrm{ET},CO_{2}}$, end‐tidal partial pressure of CO_2_; $\bar{P}$_ga_, mean inspiratory gastric pressure; $\bar{P}$_oe_, mean inspiratory oesophageal pressure; $rrP_{\mathrm{ET},CO_{2}}$, rate of rise of $P_{\mathrm{ET},CO_{2}}$; SE_M_, standard error of measurement; $\dot{V}$_I_, inspiratory minute ventilation; *V*_TI_, inspiratory tidal volume; *t*_I_, inspiratory time.

**TABLE 6:** Within‐day, between‐trial reliability of peak responses to CO_2_ rebreathing.

Parameter	Trial 1	Trial 2	SE_M_	CV (%)	ICC	*P*‐value
Ventilatory indices						
$\dot{V}$_I_, L/min	33.2 (11.0)	28.3 (8.7)	1.81	1.87 (1.06–2.69)	0.73 (−0.10–0.94)	**0.039**
*V*_TI_, L	2.38 (0.71)	2.21 (0.57)^*^	0.054	1.53 (0.86–2.19)	0.80 (0.00–0.96)	**0.008**
*f*_R_, breaths/min	14.1 (3.40)	15.0 (2.0)	0.41	2.41 (1.35–3.46)	0.46 (−0.23–0.86)	0.111
*t*_I_, s	1.85 (0.45)	2.20 (1.05)	0.232	7.41 (4.16–10.65)	0.13 (−0.61–0.73)	0.378
Inspiratory pressures						
$\;\bar{P}$_di_, cmH_2_O	13.90 (5.00)	13.80 (4.70)	0.030	0.54 (0.30–0.78)	0.82 (0.01–0.97)	**0.025**
$\;\bar{P}$_oe_, cmH_2_O	−6.80 (1.80)	−6.80 (2.50)	0.000	2.57 (1.45–3.70)	0.12 (−0.65–0.77)	0.318
$\;\bar{P}$_ga_, cmH_2_O	6.70 (4.40)	6.90 (5.10)	0.015	2.57 (1.45–3.70)	0.99 (0.94–1.00)	**<0.001**
Diaphragm shortening						
Excursion, cm	5.15 (1.29)	4.96 (1.32)	0.071	0.10 (0.06–0.14)	0.72 (0.09–0.94)	**0.018**
Excursion time, s	1.89 (0.56)	2.04 (0.92)	0.101	4.61 (2.59–6.62)	0.10 (−0.73–0.74)	0.408
Excursion velocity, cm/s	2.38 (0.60)	2.36 (0.80)	0.0076	2.11 (1.19–3.03)	0.71 (−0.06–0.94)	**0.002**
Power, cmH_2_O×cm/s	41.9 (25.5)	35.4 (21.4)	1.85	2.64 (1.49–3.80)	0.84 (0.43–0.97)	**0.002**

*Note*: Data for Trial 1 and Trial 2 are means (SD) for eight participants. Data for CV and ICC are means (95% CI). All *P*‐values reflect statistical testing of the ICCs against zero reliability, with values in bold representing *P* ≤ 0.05.

* Significant difference vs. Trial 1 (*P* ≤ 0.05).

Abbreviations: CV, coefficient of variation; *f*_R_, respiratory frequency; ICC, intraclass correlation coefficient; $\bar{P}$_di_, mean inspiratory transdiaphragmatic pressure; $\bar{P}$_ga_, mean inspiratory gastric pressure; $\bar{P}$_oe_, mean inspiratory oesophageal pressure; SE_M_, standard error of measurement; *t*_I_, inspiratory time; $\dot{V}$_I_, inspiratory minute ventilation; *V*_TI_, inspiratory tidal volume.

CO_2_ rebreathing elicited a linear increase in $\dot{V}$_I_ relative to $P_{\mathrm{ET},CO_{2}}$, with no between‐trial differences in slope or intercept. Ventilatory increases were driven by *V*_TI_ and *f*_R_, the latter facilitated by a progressive shortening of *t*_I_. Increases in $\bar{P}$_di_ were attributable primarily to reductions in $\bar{P}$_oe_, with smaller contributions from $\bar{P}$_ga_. No between‐trial differences were observed for $\bar{P}$_di_ slope (*P* = 0.937) or intercept (*P* = 0.075). At peak, $\dot{V}$_I_ reached ∼2.5 times resting values (∼20% of maximal voluntary ventilation).

#### 3.2.2 Technical feasibility

**FIGURE 5:**
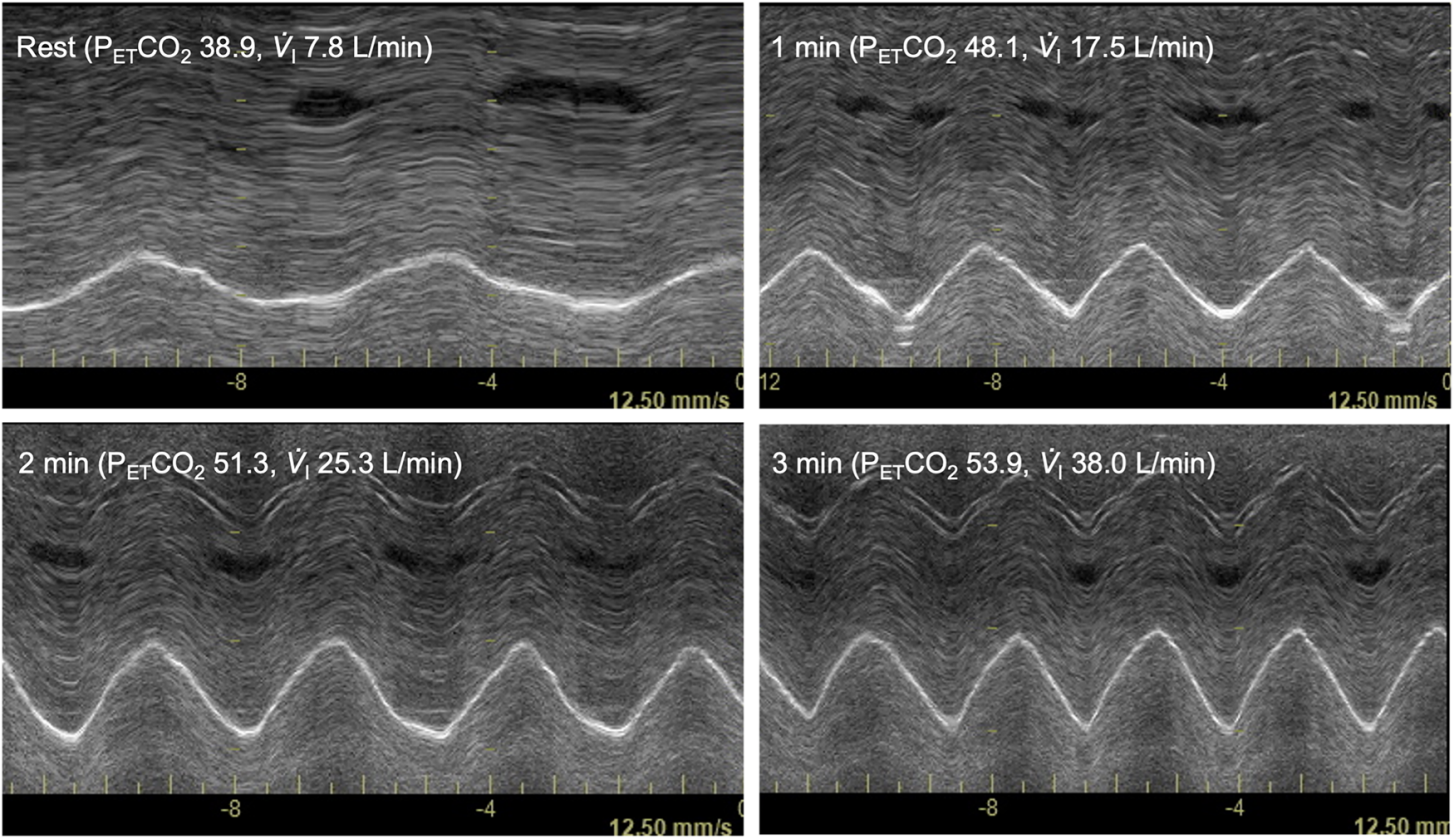
Representative anatomical motion‐mode (AM‐mode) ultrasound images of the right crural hemidiaphragm during quiet breathing (rest) and after 1, 2 and 3 min of CO2 rebreathing in a female participant. Diaphragm excursion increased progressively with rising ventilation (1.81 cm at rest; 4.89 cm at 3 min). Axis scales are identical across panels. Abbreviations: $P_{\mathrm{ET},CO_{2}}$, end‐tidal partial pressure of CO_2_; $\dot{V}$_I_, inspiratory minute ventilation.

Of 206 cine‐loop images acquired, 204 (99%) were analysable, indicating a high success rate across the full range of ventilatory responses. Representative AM‐mode ultrasound images during the CO_2_‐rebreathing protocol are shown in Figure 5.

#### 3.2.3 Construct validity

**FIGURE 6:**
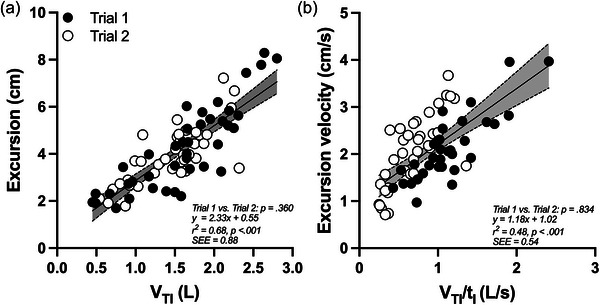
Relationships between ventilatory variables and ultrasound‐derived indices of diaphragm shortening during CO_2_ rebreathing across two trials (*n *= 8 participants). Data are shown with pooled regression lines and shaded 95% confidence intervals. Abbreviations: *t*_I_, inspiratory time; *V*_TI_, inspiratory tidal volume.

Changes in diaphragm shortening relative to $P_{\mathrm{ET},CO_{2}}$ are summarized in Table 5. From rest to peak, diaphragm excursion increased 1.6‐fold (from 3.14 ± 1.59 to 5.06 ± 1.30 cm, *P* < 0.001), excursion velocity increased 1.5‐fold (from 1.65 ± 0.53 to 2.37 ± 0.70 cm/s, *P* < 0.001) and diaphragm power increased 3.2‐fold (from 12.2 ± 9.9 to 38.7 ± 24.7 cmH_2_O×cm/s, *P* = 0.003). Excursion time remained unchanged (1.90 ± 0.67 vs. 1.95 ± 0.72 s, *P* = 0.667). At peak, diaphragm excursion reached ∼75% of maximal values (Table 1). Significant correlations were observed between excursion and *V*_TI_ (*r* = 0.83, *P* < 0.001) and between excursion velocity and *V*_TI_/*t*_I_ (*r* = 0.69, *P* < 0.001), each with small standard errors (Figure 6).

#### 3.2.4 Test–retest reliability

Ventilatory indices demonstrated ‘moderate to good’ reliability, with ICCs ranging from 0.64 to 0.73 across variables. Pressure‐derived measures showed ‘good’ reliability (ICCs = 0.81–0.82). Ultrasound‐derived indices were more reliable for peak responses than slope measures: peak excursion, velocity and power achieved ‘moderate to good’ reliability (ICCs = 0.71–0.84), whereas slope‐based responses showed ‘poor’ reliability (ICCs ≤ 0.20) and were associated with wide confidence intervals.

## 4 DISCUSSION

This study is the first to evaluate the combined use of subcostal ultrasonography and respiratory manometry to characterize the contractile properties of the human diaphragm across non‐volitional, volitional and reflexive perturbations. The main findings were as follows: (1) ultrasound acquisition and analysis were technically feasible across a wide range of experimental conditions; (2) diaphragm contractile function was systematically modulated by postactivation potentiation, stimulation frequency and voluntary effort, thereby supporting the construct validity of ultrasound‐derived indices; (3) test–retest reliability was enhanced by potentiation, high‐frequency stimulation and voluntary efforts, with ultrasound‐derived indices generally exhibiting superior reliability compared with pressure‐based measures; and (4) CO_2_ rebreathing elicited predictable increases in diaphragm excursion, velocity and power that closely paralleled ventilatory and pressure responses, with peak‐based indices demonstrating greater reliability than slope‐derived measures. Collectively, these findings support the feasibility, validity and reliability of this integrated method for comprehensive assessment of diaphragm contractile function.

### 4.1 Feasibility

Subcostal ultrasonography was technically feasible for quantifying diaphragm motion in response to all perturbations examined, including unilateral phrenic nerve stimulation, maximal voluntary sniffs and CO_2_‐induced hyperpnoea. Imaging failures during nerve stimulation were infrequent and occurred primarily when the right hemidiaphragm moved laterally beyond the ultrasound field of view, consistent with asymmetric mechanical coupling between the right and left hemidiaphragms during unilateral activation (Bellemare et al., 1986; De Troyer et al., 2003). Additional difficulties in image acquisition were attributable to participant‐specific anatomical characteristics, particularly large thoracic dimensions, whereby increased chest circumference and thoracic wall thickness increase the probe‐to‐diaphragm distance and degrade image quality. Nevertheless, the high imaging success observed across all conditions confirm the technical feasibility of subcostal ultrasonography for assessing diaphragm motion.

### 4.2 Validity

Diaphragm excursion, excursion time and excursion velocity during quiet breathing and maximal sniffs were within established normative ranges (Boussuges et al., 2020; Dres et al., 2025; Laghi et al., 2021). Importantly, synchronous integration of subcostal ultrasonography with pressure measurements enabled a comprehensive assessment of diaphragm contractile function. In the context of phrenic nerve stimulation, this integrated method represents a substantive advance over earlier studies that either did not report pressure or excursion measurements (McCauley & Labib, 1984; Mills et al., 1995) or obtained ultrasound and manometry data during separate manoeuvres (Spiesshoefer et al., 2019, 2020).

The increase in stimulation‐evoked *P*_di_ following prior voluntary contraction is consistent with postactivation potentiation (Mador et al., 1994; Wragg et al., 1994). This increase in twitch *P*_di_ was accompanied by proportional increases in diaphragm excursion and, to a lesser extent, excursion velocity. The primary mechanism underlying postactivation potentiation is phosphorylation of the myosin regulatory light chain, which increases the sensitivity of the actin–myosin complex to calcium and thereby facilitates enhanced cross‐bridge binding at submaximal intracellular calcium concentrations (Manning & Stull, 1979; Persechini et al., 1985). This biochemical modification increases the rate of cross‐bridge cycling, resulting in faster force development and increased contractile velocity (Bowslaugh et al., 2016; Gittings et al., 2012; MacIntosh & Bryan, 2002). Additional contributions from reductions in series‐elastic compliance may further augment contractile performance. Collectively, these mechanisms favour a disproportionate increase in power output relative to force alone, consistent with the ultrasound‐derived metrics reported here.

Paired unilateral phrenic nerve stimulation elicited frequency‐dependent increases in *P*_di_, excursion, velocity and power, with excursion reaching a plateau at 50 Hz. At lower frequencies (1–10 Hz), excursion velocity and power remained relatively stable, likely reflecting insufficient temporal summation due to prolonged interstimulus intervals. At higher frequencies, both velocity and power increased markedly as a consequence of shortened excursion time. Unlike bilateral stimulation, where *P*_di_ typically plateaus above 20–30 Hz (Babcock et al., 1998; Yan et al., 1993), no plateau was observed during unilateral stimulation, likely reflecting the reduced mechanical load associated with asymmetric activation. The resulting frequency–response profile is consistent with preferential recruitment of fast‐twitch fibres and a shift towards a velocity‐optimized region of the diaphragm force–velocity relationship (Coirault et al., 1997; Fournier & Sieck, 1988). These findings highlight the sensitivity of ultrasonography to detect contractile adjustments that might otherwise be obscured when relying solely on pressure‐based indices.

During CO_2_ rebreathing, diaphragm excursion and velocity increased systematically with hypercapnia and correlated significantly with inspiratory tidal volume and mean inspiratory flow, respectively. However, these ventilatory variables accounted for <70% of the total variance in ultrasound‐derived indices, reflecting both measurement variability and the inherent complexity of crural mechanics (Newman et al., 1984). Localized ultrasonographic measurements are influenced by regional heterogeneity in fibre recruitment, length–tension relationships and the dynamic interaction between diaphragm motion and thoracoabdominal compliance. Moreover, reflexive modulation of neural drive during progressive hypercapnia may alter motor‐unit recruitment strategies, producing contractile behaviours not fully captured by flow‐based metrics. Recruitment of accessory respiratory muscles and inter‐individual differences in diaphragm activation, particularly in the absence of participant coaching, probably likely further contribute to this variability. Together, these observations reinforce the value of ultrasonography for providing mechanistic insight into diaphragm contractile function under non‐volitional, reflex‐mediated conditions.

### 4.3 Reliability

For single‐twitch contractions evoked by unilateral phrenic nerve stimulation, within‐day CVs for non‐potentiated *P*_di_ (1.9%) and potentiated *P*_di_ (4.4%) were comparable to previously reported values for bilateral magnetic stimulation [non‐potentiated: 5.1% (Mador et al., 2002) or 4.5% (Taylor et al., 2010); potentiated: 5.6% (Taylor & Romer, 2009), 2.9% (Taylor et al., 2010) or 7.3% (Tiller et al., 2017)]. Based on CVs alone, non‐potentiated twitches appeared marginally more reliable; however, this pattern was reversed when considering ICCs, likely reflecting low between‐participant variability within this homogeneous cohort, which is a recognized limitation of ICC interpretation (Atkinson & Nevill, 1998). The ICC for potentiated *P*_di_ during unilateral stimulation (0.63) was also lower than that reported for bilateral cervical stimulation (0.89; Ramsook et al., 2021), indicating reduced reliability under unilateral conditions. Nevertheless, the low CVs and close agreement with published values support unilateral twitch *P*_di_ as a reliable within‐participant index of diaphragm function.

Ultrasound‐derived indices generally exhibited superior reliability to pressure‐based measures, particularly for higher‐amplitude responses. Potentiated twitches and high‐frequency stimulations (50–100 Hz) demonstrated excellent reliability, with excursion velocity achieving an ICC of 0.94. Maximal voluntary sniffs were also highly reliable, particularly for excursion time (ICC = 0.94) and power (CV = 0.11%). These findings suggest that high‐intensity contractions improve measurement precision by enhancing signal resolution and increasing between‐participant variability.

The high reliability observed in this study likely reflects standardized procedures, participant familiarization and sonographer expertise. All participants completed a dedicated familiarization session, and ultrasound data were acquired by a single experienced sonographer who had completed >40 examinations, i.e., the recommended minimum for proficiency in diaphragm ultrasonography (Haaksma et al., 2022). Image acquisition followed a standardized protocol with fixed transducer placement based on anatomical landmarks, consistent depth and gain settings, and controlled timing within the respiratory cycle. Use of angle‐independent M‐mode imaging and objective analysis criteria further minimized operator‐related variability.

Methodological differences may also explain discrepancies with previous work. Pilot data from Poulard et al. (2020) suggested highly variable diaphragm excursion responses to cervical magnetic stimulation, potentially reflecting limited participant familiarization and inherent constraints of the cervical approach (Angus et al., 2023). Notably, only 2 of 13 participants in that study achieved maximal phrenic nerve activation. In contrast, unilateral anterolateral stimulation in the present study consistently elicited maximal activation, as confirmed by a plateau in twitch *P*_di_ during incremental stimulation in all participants (see Materials and Methods).

For the CO_2_‐rebreathing protocol, reliability varied by outcome. Slope‐based indices demonstrated poor reliability (ICCs ≤ 0.20), despite relatively low variability in the overall ventilatory response (∼10%) compared with previous studies [26% (Jensen et al., 2010); 18% (Sahn et al., 1977)]. This likely reflects the sensitivity of slope measures to small fluctuations in chemosensitivity and breathing pattern. In contrast, peak‐response indices exhibited good to excellent reliability (ICCs = 0.71–0.84) and are therefore more suitable for between‐participant comparisons, whereas slope measures may remain useful for within‐participant monitoring of acute interventions.

### 4.4 Limitations and future directions

Severeal limitations should be acknowledged. First, participants were young, healthy adults with an uneven sex distribution, limiting generalizability. Although relative responses and reliability were comparable between sexes, males exhibited higher absolute values (consistent with established sex differences in diaphragm function), highlighting the need to evaluate females, older adults and clinical populations to establish normative reference ranges. Second, assessments were confined to the diaphragm; extradiaphragmatic respiratory muscles may exhibit distinct contractile behaviour and warrant investigation. Third, only within‐day reliability was assessed; between‐day reliability is required to confirm suitability for longitudinal monitoring. Fourth, ultrasonography is inherently operator dependent and sensitive to anatomical variability. Although variability was minimized through use of a single experienced operator, angle‐independent M‐mode imaging and standardized acquisition protocols, full blinding during analysis was not feasible, and prior knowledge of participant data may have introduced bias, albeit mitigated by objective measurement criteria. Fifth, temporal indices of contraction (e.g., time to peak pressure, half‐relaxation time) were not evaluated but may provide additional mechanistic insight. Finally, advanced ultrasound techniques (e.g., tissue Doppler imaging, shear‐wave elastography, speckle tracking), particularly when integrated with complementary assessments such as diaphragm electromyography, may further enhance characterization of neuromechanical function and should be explored in future studies.

## 5 CONCLUSION

Subcostal ultrasonography combined with respiratory manometry is a feasible, valid and reliable method for assessing diaphragm contractile function across evoked, volitional and reflexive perturbations. Excursion velocity and power emerged as the most robust and sensitive indices, particularly during potentiated, high‐frequency or voluntary contractions. Peak‐response metrics were more reliable than slope‐based measures, and real‐time integration of pressure and motion enables evaluation of force–velocity coupling. Future studies should prioritize these indices and experimental conditions to optimize measurement reliability, facilitate repeated‐measures designs and enhance mechanistic interpretation. In clinical and translational contexts, this method may support early detection of diaphragm dysfunction, monitoring of recovery and evaluation of interventions. By capturing both force output and shortening dynamics, this integrated method provides a comprehensive framework for investigating diaphragm function in health and disease.

## AUTHOR CONTRIBUTIONS

The experiments were performed at Brunel University of London. Camilla R. Illidi and Lee M. Romer conceived and designed the study, and both were involved in data collection, analysis and interpretation. Camilla R. Illidi and Lee M. Romer drafted the manuscript and revised it critically for important intellectual content. Both authors approved the final version of the manuscript and agree to be accountable for all aspects of the work in ensuring that questions related to the accuracy or integrity of any part of the work are appropriately investigated and resolved. Both persons designated as authors qualify for authorship, and all those who qualify for authorship are listed.

## CONFLICT OF INTEREST

None declared.

## FUNDING INFORMATION

None.

## Data Availability

The data that support the findings of this study are shown in the figures, tables and supporting information.
